# Development of an expectation management intervention for patients with Long COVID: A focus group study with affected patients

**DOI:** 10.1371/journal.pone.0317905

**Published:** 2025-02-03

**Authors:** Manuel Funk, Max Reinke, Bernd Löwe, Petra Engelmann

**Affiliations:** Department of Psychosomatic Medicine and Psychotherapy, University Medical Center Hamburg-Eppendorf, Hamburg, Germany; Medical University of Vienna, AUSTRIA

## Abstract

**Background:**

A significant number of individuals who have contracted SARS-CoV-2 report persistent somatic symptoms after the infection has resolved. Evidence-based treatment options for Long COVID are lacking to date. To ensure that an expectation management intervention, designed for the research project SOMA.COV, addresses relevant patient needs as well as to promote treatment acceptance and adherence, a participatory approach was chosen.

**Objective:**

The aim of the present study was to explore needs and wishes of patients with Long COVID regarding the preliminary version of an expectation management intervention and to thereby inform the further development of the treatment manual.

**Methods:**

Twenty-two patients affected by Long COVID participated in one of four focus groups in June and July 2023. Participants were presented with the draft content of a four-session expectation management intervention. Feedback was audio-recorded, transcribed, and analyzed using thematic analysis.

**Results:**

Thirteen themes relating to the main components of the intervention manual were developed. Large parts of the manual received overall positive feedback, including psychoeducation on the biopsychosocial etiology of the condition, elements of cognitive restructuring, and an imagination exercise. Patients’ response to the presented vicious circle of fear and a behavior change exercise was mixed. Modifications to the manual were made in response to patients’ feedback.

**Conclusion:**

Patients with Long COVID provided positive feedback on an expectation management intervention while also highlighting important adaptations necessary for this patient group. The study results informed the finalization of the treatment manual within the SOMA.COV project, which investigates the effectiveness of this intervention for patients with Long COVID in a three-armed randomized controlled trial.

## Introduction

Recent studies on the natural course of Long COVID [1] suggest that a majority of those affected continue to experience somatic symptoms such as fatigue, cognitive deficits, and pain for up to more than one year after SARS-CoV-2 infection [2,3]. Although immunological, virological, vascular, and neurological findings are discussed, the pathophysiology of Long COVID remains largely unclear and causal treatment options addressing the underlying pathomechanisms of the condition are missing [4]. Recent research identified biological risk factors for the development and maintenance of Long COVID – such as female gender, older age, and disease severity in the acute phase of COVID-19 [5] – and social risk factors like loneliness [6], less social support, and financial hardship [7]. In addition, several studies also highlight the role of psychological risk factors for the development and maintenance of Long COVID [6,8]. Altogether, this points to a complex biopsychosocial etiology of the condition similar to other persistent somatic symptoms and chronic medical conditions [9–11]. While most evidence on psychological risk factors confirms the importance of (illness-related) anxiety and depression [12–14], other psychological variables such as dysfunctional expectations, i.e., future-directed beliefs of increased frequency or intensity of certain symptoms [15], also seem to contribute to symptom persistence after COVID-19 [16,17]. Psychological interventions aiming to modify patient expectations via elements of cognitive behavioral therapy (CBT) have shown to improve clinical outcomes in several medical conditions, including coronary heart disease, cancer, and chronic pain [18,19]. Despite controversy regarding the effectiveness of CBT interventions for other fatigue-related conditions such as myalgic encephalomyelitis/chronic fatigue syndrome (ME/CFS) [20], first interventional studies addressing psychological factors in using CBT have shown effective in reducing fatigue in patients with Long COVID [21,22]. However, therapeutic approaches specifically targeting symptom expectations and illness-related anxiety in Long COVID are lacking.

Accordingly, our ongoing research project SOMA.COV [23] investigates the effect of a newly developed treatment approach aiming at the modification of both factors on Long COVID symptoms. The expectation management intervention consists of four individual online sessions. In a preliminary version of the intervention manual, the first three sessions consisted of psychoeducation on the biopsychosocial model [9,10], cognitive restructuring of dysfunctional symptom expectations, an imagination exercise aiming to induce positive expectations, and education on perpetuating factors in the vicious circle of fear (acc. to [24]). At the end of the third session, a behavior change exercise was planned in which the degree of conviction, the context of the exercise and the avoidance behavior that is planned to be discontinued are specified in a written form. The fourth session was intended as a booster session (for further information see [23] and S1 File).

Since knowledge about effective therapies for Long COVID is still highly limited, engaging patients in the development of treatments plays a pivotal role for meeting the needs of those affected and enhancing treatment adherence [25]. This seems to be particularly important for patients with Long COVID, who often feel abandoned and experience a lack of support from health care providers [26]. The fact that psychosocial mechanisms are often neglected in current explanatory models for Long COVID [27] increases the need to explore acceptability of a biopsychosocial perspective among those affected by the condition. However, a systematic review of registered trials on Long COVID found that so far only few studies have involved patients with Long COVID in intervention development [28]. Examples exist for the development of a integrative, manualized psychotherapeutic intervention in Long COVID [29] as well as of self-management support [30]. Besides, research studies on patients’ evaluations of existing psychosocial interventions remain scarce [31].

Therefore, the aim of this qualitative study was to actively involve patients with Long COVID in the further development of the SOMA.COV expectation management intervention manual using focus group interviews. In particular, we aimed to explore patients’ lived experiences, needs, and perspectives on the draft of the intervention manual in order to subsequently adapt the intervention based on patients’ feedback.

## Methods

### Study design

The present qualitative focus group study was part of the research project SOMA.COV (“Long COVID: Psychological risk factors and their modification”), an observer-blinded, three-arm, randomized controlled trial aiming to compare the effect of an expectation management intervention for patients with Long COVID to an unspecific supportive intervention and treatment as usual only [23]. SOMA.COV is associated with the interdisciplinary research unit SOMACROSS (RU 5211) [10].

In this study, adult patients who suffered from Long COVID were presented with the manual of the SOMA.COV expectation management intervention and their feedback was obtained in focus group interviews in order to optimize the content of the intervention according to the needs of those affected. A brief summary of the intervention manual can be found in the S1 File.

### Participants and Setting

The study participants were selected via convenience sampling and comprised outpatients who visited an eight-session CBT-based group therapy for Long COVID at the psychosomatic outpatient clinic of the University Medical Center Hamburg-Eppendorf, Germany (UKE), as well as members of a Long COVID support group from Lower Saxony, Germany. Inclusion criteria were resolved SARS-CoV-2 infection, self-reported history of Long COVID according to the NICE guideline [1], age ≥  18 years, and provision of written informed consent. Patients with insufficient knowledge of German language were excluded. Recruitment was carried out between 25^th^ of May until 27^th^ of June 2023, and the focus groups were conducted between in June 2023. Eligible patients were informed about the study and invited to participate via telephone calls. In case participants verbally agreed to participate, they received a written declaration of consent and a privacy policy by mail. Participation in the study was reimbursed at 15 euros per hour. The inclusion of participants was carried out until empirical saturation was reached, which means that no further new and relevant content was identified.

### Data collection

A semi-structured interview guide consisting of 17 open-ended questions which related to the individual sessions of the manual and to general feedback was first developed by three authors (MF, MR, PE) and then revised based on suggestions for improvement of a group of researchers (physicians and psychologists) working at the Department of Psychosomatic Medicine and Psychotherapy at the UKE. The interview guide can be found in the S2 File.

Initial questions from the guide related to lived experiences and coping with the illness as well as previous treatment experiences and treatment needs. In this way, an overview of individual patient experiences with Long COVID was gained. The results regarding these topics will be published elsewhere. After presentation of the intervention manual, subsequent questions focused on the comprehensibility of the biopsychosocial model and the role of symptom expectations and illness-related anxiety for symptom persistence from patients’ perspective as well as the feasibility of the imagination exercise, which was performed in full length in all focus groups. In addition, questions relating to the comprehensibility and applicability of the vicious circle of fear and the behavior change exercise were asked. At the end, final feedback was obtained, including suggestions for additions to the manual.

Overall, four focus groups of three hours each took place, with four to seven patients in each group. Three focus groups were held on the premises of the psychosomatic outpatient clinic at the UKE and one focus group took place online. In addition to two interviewers (MF and MR) and the study participants, a student assistant was present in each focus group to take notes on the participants’ statements. To characterize the study sample, sociodemographic (sex, age, employment) and Long COVID-related information (number of SARS-CoV-2 infections, presence and duration of Long COVID) was assessed prior to the interviews. Persistent somatic symptoms were assessed with the Patient Health Questionnaire-15 (PHQ-15), which measures burden by fifteen common somatic symptoms (e.g., pain, fatigue, and shortness of breath) within the last four weeks on a three-point scale (from “not bothered at all” to “bothered a lot”). Sum scores are categorized into minimal (1-4 points), low (5-9 points), moderate (10-14 points), and severe (15-30 points) somatic symptom burden. Internal consistency of the PHQ-15 can be considered high (Cronbach`s α 0.80) [32].

Interview data was collected using audio-recordings and transcribed verbatim by two student assistants and two authors (MF, MR) while assuring pseudonymization [33]. Transcripts were created using the software MAXQDA (MAXQDA Analytics Pro 2022 Network, VERBI), which was subsequently used to analyze the data. Transcripts were not given to the participants for correction.

## Data analysis

Thematic Analysis by Braun and Clarke was used in six steps: (1) familiarizing with the data, (2) generating codes, (3) combining codes into themes, (4) reviewing themes, (5) defining and naming themes, and (6) reporting [34]. The first step consisted of taking notes on the participants’ statements to obtain an initial overview. In line with collaborative coding [34], all four focus groups transcripts were then coded twice by two authors (MF, MR) to promote diversity of interpretation. During the analysis, the focus was placed on a semantic and therefore mainly explicit level. The analysis enabled a combined inductive and deductive approach to identify key topics relating to the research questions [35]. On the one hand, new themes were developed in a relatively free manner in the course of coding the data (inductive). On the other hand, specific evaluation questions were asked along the structure of the intervention (deductive). A codebook was created to facilitate the analysis process. In the next step, the codes were combined into initial themes. The codebook is available upon request from the corresponding author. MF and MR were in regular discussion with the head psychologist (PE). These meetings led to new themes with new emphases and definitions until all three researchers agreed on a final identification of themes. Results were not made available to the participants at the end and were therefore not discussed with them. Reporting of the results is based on the checklist for “Consolidated Criteria for Reporting Qualitative Research” (COREQ; see S3 File) [36].

### Research team and reflexivity

The focus groups were conducted by two authors: MF, physician, and MR, psychologist with a master’s degree and experience in qualitative research. Both interviewers were male and in training as cognitive behavioral psychotherapists. The study was supervised by PE (female), project manager of SOMA.COV and head psychologist of the Department of Psychosomatic Medicine and Psychotherapy at the UKE with a PhD degree. At the time of the study, MF was working in the outpatient clinic of the same department and already knew four participants from group psychotherapy sessions there. Personal information about the researchers was not known to the participants.

### Ethical considerations

The present study was approved by the Local Psychological Ethics Committee (LPEK) at the Center for Psychosocial Medicine of the University Medical Center Hamburg-Eppendorf on May 23rd, 2023 (approval number LPEK‐0624). The study adheres to the guidelines of the World Medical Association Declaration of Helsinki from 1964 as amended in 2013 [37].

## Results

Initially, 30 patients who met the inclusion criteria were contacted. Eight patients could either not be reached or declined participation. This resulted in a total of *N* =  22 patients included in the study. Of those, seven patients took part in the first focus group, five in the second online group, four in the third group, and six in the fourth group. Participants were aged between 28 and 63 years, with the majority being female, and exhibited a moderate mean somatic symptom burden. Sociodemographic characteristics, information related to Long COVID, and results of the PHQ-15 are presented in Table 1.

**Table 1:** Sociodemographic and clinical characteristics of the study group (*N* =  22).

Variable	Study sample
Sex, *n* (%)	
Female	17 (77.3%)
Male	4 (18.2%)
Diverse	1 (4.6%)
Age, *M* (*SD*)	43.6 (11.55)
Living situation, *n* (%)	
Living together	16 (72.7%)
Living alone	4 (18.2%)
Education, *n* (%)	
Without professional qualification	0 (0%)
In vocational training	0 (0%)
Completed vocational training	14 (63.6%)
University degree	8 (36.4%)
Employment, *n* (%)	
Currently not working	10 (45.5%)
Retired	1 (4.5%)
Unemployed	3 (13.6%)
Employed part-time	6 (27.3%)
Employed full-time	2 (9.1%)
Number of SARS-CoV-2 infections, *n* (%)	
1	13 (59.1%)
2	5 (22.7%)
3	3 (13.6%)
4	1 (4.6%)
Long COVID status, *n* (%)	
Recovered	1 (4.5%)
Still affected	21 (95.5%)
Months since self-reported Long COVID, *M* (*SD*)	19.4 (10.5)
PHQ-15, *n* (%)	
Minimal	1 (4.5%)
Low	3 (13.6%)
Moderate	8 (36.4%)
Severe	10 (45.5%)
PHQ-15, *M* (*SD*)	14.7 (4.98)

Note. *n* =  number of participants; *M* =  mean; *SD* =  standard deviation; PHQ-15 =  Patient Health Questionnaire-15.

In the course of data analysis, we developed 13 themes (see Table 2) that reflect participants’ statements on the six main topics of the interventional manual. Characteristic quotes were selected and translated from German into English.

**Table 2:** Overview of developed themes related to the six topics of the expectation management intervention manual of the SOMA.COV trial.

Topic	Themes
1: Biopsychosocial model (1^st^ session)	Agreement on the interaction between body and mind
2: Cognitive restructuring (2^nd^ session)	“Change in the mind”: Opportunities for cognitive restructuring
	“Plan B”: Acceptance and reorientation as an additional cognitive approach
3: Imagination exercise (2^nd^ session)	“Cartwheels”: Unfolding vitality and energy
	“Less pain”: Pleasant body sensation
	“Bubbling kettle”: Concentration difficulties and challenges in the transfer to everyday life
4: Vicious circle of fear (3^rd^ session)	“Snail shell”: Avoidance as a consequence of fear
	“Calculation”: Risk assessment instead of fear
	“Illness of dashed hopes”: Self-overload instead of avoidance
5: Behavior change exercise (3^rd^ session)	Promotion of self-confidence: Encouragement to carry out the exercise
	Criticism of the terms “avoidance behavior” and “result”
	Determining the framework conditions: Additional suggestions
6: Further feedback and needs	“Pacing” and provision of resources: Participants’ wishes

## Topic 1: Biopsychosocial model

The first topic deals with feedback on the comprehensibility of the presented biopsychosocial model that is part of the first session of the intervention.

### Agreement on the interaction between body and mind

Overall, the introduction to the general biopsychosocial model was rated as comprehensible and easy to adapt by the majority of participants. The interviewees appeared to find it easier to recognize the influence of physical conditions and social factors on the psyche than vice versa.

Many participants emphasized that their somatic symptoms of Long COVID are connected to their emotions and thoughts and gave examples of this. Especially, depressed mood, anxiety, and desperation were mentioned several times as consequences of physical impairment and no improvement in health status:

“When the body functions properly, the mind often functions better as well.” (P2)

“You are disappointed in yourself, because you do so much. Why the hell doesn’t it get any better?” (P16)

The impact of social factors – such as stress in the family, challenging work or financial situations or difficulties with authorities – on mental and physical health, including sleeping behavior, was underlined by a few participants.

“All the drama with the authorities has been going on for two and a half years, so I can’t rest. The health insurance company is constantly pressuring me.” (P3)

“My adolescent son is still running around at 11 o’clock in the evening in an extremely bad mood. This is preventing me from sleeping and causing me stress.” (P10)

One participant could not relate to the biopsychosocial model, and rated anxiety and sadness as largely independent of processes in the body: “No, I don’t think it affects the body.” (P17)

## Topic 2: Cognitive restructuring

The second topic comprises patients’ perspective on the role of dysfunctional cognitions (symptom expectations and anxious thoughts in the context of illness-related anxiety) in Long COVID, which are addressed as part of cognitive restructuring in the second session.

### “Change in the mind”: Opportunities for cognitive restructuring

Almost all participants confirmed that functional thoughts, such as those proposed within the intervention, promote a balanced mood and behavioral activation. Modifying negative expectations was experienced as a helpful coping strategy:

“I changed my mind suddenly and thought ‘Now I am going to make it’. And indeed, things are improving now.” (P3)

“I have the expectation that it [reading] will work again soon, so I am practicing.” (P14)

Another helpful approach that was frequently mentioned was the practice of shifting one’s attention to one’s own progress, such as extending one’s running route, or focusing on the progresses of other patients: “I focused more on the people who have found a way.” (P3)

### “Plan B”: Acceptance and reorientation as an additional cognitive approach

Some participants reported that the acceptance of having to adapt activities such as housework and hobbies to their condition boosted their confidence and self-efficacy, which was beneficial in dealing with Long COVID. Although hobbies could no longer be pursued with the same intensity due to rapid exhaustion, they tried to accept these life changes. One patient reported having found joy in the creativity required to reorganize activities:

“I trust in having a plan B. When I am unable to proceed, I reflect on what I had enjoyed in the past and consider alternative ways to achieve it. Being creative can be enjoyable.” (P1)

“When I overload myself, I acknowledge it and try to accept it: ‘Okay, I have totally overloaded myself and that is the reality now.’” (P10)

## Topic 3: Imagination exercise

The third topic summarizes feedback on the feasibility of the imagination exercise, which aims to improve body image and induce positive expectations and was carried out with all participants as further part of the second session.

### “Cartwheels”: Unfolding vitality and energy

The majority of patients reported that imagining a positive body image activated satisfying feelings such as lightness, liveliness, and joy. They stated to have been able to engage well with the exercise and described positive images that the exercise had evoked. Imaginations in which participants were able to perform activities in a way that was currently not possible in real life were well received. Some participants felt so comfortable that they were reluctant to end the exercise:

“I felt energized and active. I pictured myself in a sunny stubble field, doing cartwheels and somersaults. It was a delightful experience. I didn’t worry about it not working anymore, but rather enjoyed the image as if I was actually doing it.” (P1)

“I just strolled through the day, just as I am, under trees, slightly shady.” (P7)

### “Less pain”: Pleasant body sensation

The image of being symptom-free was emphasized as a novel, very profitable experience. One patient reported to even have felt less pain during the exercise:

“I didn’t think about my main symptoms, which are annoying, and that was a pleasant feeling.” (P3)

“The idea of being carefree, of simply living without feeling anything in your body that hurts, just like before, was born.” (P17)

### “Bubbling kettle”: Concentration difficulties and challenges in the transfer to everyday life

A couple of participants acknowledged negative experiences during the imagination exercise, such as difficulties concentrating. One interviewee mentioned feeling stressed and tense in response to the exercise, particularly due to the requirement to actively imagine bodily sensations:

“Staying focused is stressful. It feels like a kettle starting to bubble, creating tension instead of relaxation.” (P16)

One participant emphasized the importance of guided instruction in dealing with his pain. He believed that the exercise would not be helpful in the long term due to difficulties in performing it independently without guidance in his everyday life:

“But if I’ll do it [the exercise] on my own, it won’t help me much.” (P11)

## Topic 4: Vicious circle of fear

The fourth topic comprises participants’ statements on the comprehensibility and suitability of the vicious circle of fear explained in the third session to illustrate maintenance of illness-related anxiety.

### “Snail shell”: Avoidance as a consequence of fear

Three patients reported to benefit from psychoeducation on the vicious circle of fear. For instance, they stated that excessive illness-related anxiety and the associated reinforcement of somatic symptoms caused them to cancel upcoming appointments, meetings with friends, or parties:

“I canceled all the parties and celebrations that were coming up, just out of fear that I wouldn’t be able to handle it. I withdraw like a snail and prefer to lie down and sleep.” (P19)

### “Calculation”: Risk assessment instead of fear

Most participants considered it important to emphasize that the avoidance behavior depicted in the vicious circle was not a consequence of excessive anxiety, but a “healthy response” to a “real risk assessment” (P4). In case of symptoms, it was advisable for them to avoid potential symptom exacerbation and to protect themselves from another “crash” by taking a break or planning ahead. Some interviewees reported they felt stigmatized by society and falsely accused of unjustified avoidance behavior:

“There is nothing wrong with saying ‘I’m not going to go jogging’ because I know I am going to crash afterwards. That is protection. Long COVID is not recognized as a life-threatening disease. […] I know that symptoms will occur if I do certain things.” (P6)

“I do not consciously avoid things out of fear, but rather calculate what I am capable of doing.” (P8)

### “Illness of dashed hopes”: Self-overload instead of avoidance

In all four focus groups, participants pointed out that they frequently pushed themselves to their limits when engaging in activities. If symptoms such as rapid exhaustion after a walk became less frequent, they tended to overload quickly. They reported feeling highly motivated to improve their performance and experiencing disappointment in case of failure. One participant described the “illness of dashed hopes” as constant effort with the frequent feeling of “running into a wall” (P8). Accordingly, the term “avoidance behavior” in the context of reduced activity was rated as unsuitable by most participants:

“I was annoyed and said to myself: ‘now you’re running again’. [...] And then I ended up in the ditch, I was so exhausted.” (P2)

“On good days, it is satisfying to feel like you are making progress and accomplish a lot. However, it is important not to overextend yourself and cross boundaries. Learning to hold back is crucial, even if you have the desire to do more.” (P8)

These persistent “setbacks” would lead to a discrepancy between the individual’s self-perception and their primary aspirations on the one hand and their actual disability on the other. This discrepancy would have a negative impact on the mood of some patients:

“I have always had a positive outlook on everything: ‘You can do it’. I would feel disappointed if I did not succeed.” (P16)

“I am very ambitious and performance-oriented. However, there have been times when setbacks have affected my motivation and dragged me down.” (P20)

## Topic 5: Behavior change exercise

The fifth topic captures patients’ feedback on the behavior change exercise, which aims at reducing avoidance behavior and gradually increasing activity while challenging dysfunctional symptom expectations. The exercise is part of the third session and presented on a work sheet.

### Promotion of self-confidence: Encouragement to carry out the exercise

The majority of participants reacted positively to the behavior exercise. Their own experiences seemed to undermine that it is worth trying things out again to make positive learning experiences. Frequently anticipated dysfunctional expectations of negative outcomes mostly did not come true:

“That is what I did for myself. Ultimately, I realized that I didn’t need my avoidance strategy or safety behavior because I was still able to resume after the appointment. Reflecting on and comparing this experience to my expectations was beneficial for me.” (P15)

Additionally, patients described that engaging in self-initiated activities such as sports and hobbies (e.g., creative work) could increase self-confidence and provide symptom relief. For instance, one participant mentioned that working out helped her to regain “a piece of [her] old life” (P9). Accordingly, the exercise was well received:

“After watching a five-minute YouTube tutorial [on sewing curtains], I successfully completed the task. This boosted my confidence and self-efficacy.” (P7)

“I experienced that I had overcome avoidance behavior and strengthened my self-confidence as a result.” (P13)

Many patients perceived a worksheet on which the person’s individual anxieties and framework conditions of the exercise are written as helpful to better recall what they had planned and to visualize changes when repeating the exercise. One participant compared this documentation to a “stress diary” in everyday life (P9) and highlighted the advantage of tracking progress and reflecting on obstacles.

### Criticism of the terms “avoidance behavior” and “result”

Negative feedback related to the wording of two parts of the worksheet. First, it was important to some participants that the choice of words did not create any “performance expectations” (P4). Since the exercise focusses on trying out formerly avoided activities rather than on “performing”, they proposed to rephrase the term “result” in a more open and future-oriented way. Suggestions included “learning process” (P5) or “[w]hat will be possible next time?” (P20).

Similar to the vicious circle, many participants criticized the phrase “avoidance behavior” and in some cases considered it offensive. Compared to anxiety disorders, some patients did not regard inactivity as a dysfunctional coping strategy but as a self-protection. Other patients compared Long COVID to other chronic conditions such as diabetes, thereby reflecting on the reasonableness of their concerns:

“Any person with diabetes who says ‘I cannot attend a cake festival because I am afraid of consuming too much sugar’ is making a wise decision.” (P6).

### Determining the framework conditions: Additional suggestions

Few patients expressed the wish to include potential precautions, such as preparations the day before, into the planning of the behavior change exercise. Planning for different scenarios was not perceived as dysfunctional safety behavior, but as very essential due to risk assessment:

“It is very important to establish clear framework conditions before attempting any action.” (P4)

“When I’m invited, I wash my hair the night before because it is too stressful to do it on the same day. It may be helpful to prepare if you have something planned.” (P17)

## Topic 6: Further feedback and needs

The last topic deals with general feedback on the intervention manual with a focus on further suggestions for changes and additions.

### “Pacing” and provision of resources: Participants’ wishes

Many participants mentioned “pacing”, the restriction of activities based on subjective energy levels [38], as a strategy for coping with Long COVID. They reacted surprised to the concept not being included in the manual and suggested addressing it in the intervention:

“I would be totally surprised if this topic [pacing] didn’t come up at all.” (P1)

Some participants expressed a desire for additional resources to be included in the intervention. These resources could include support for effectively communicating personal boundaries to family, friends, and others, as well as an “interest list” (P7), a list of positive activities, to help cope with frustration and somatic symptoms:

“I missed how to communicate changes to your friends, family, or acquaintances. Some people react with understanding while others are defiant and cannot accept that things are changing for them as well.” (P5)

“It is crucial to find empowering resources to help cope with frustration and strengthen the motivation to deal with the illness.” (P8)

### Changes to the manual of the expectation management intervention based on patients’ feedback

Based on patients’ feedback in the focus group interviews, most adjustments were made to the third session. To promote patients’ identification with the material and to increase treatment adherence, the “vicious circle of fear” was replaced by the “vicious circle of protective behavior” and the term “avoidance behavior” was removed. The behavior change exercise underwent several transformations: The main focus of the exercise was shifted to testing one’s negative symptom expectations instead of overcoming avoidance behavior and the conditions for carrying out the exercise were expanded and clarified (e.g., place and time of the exercise). An open outcome was emphasized to alleviate pressure to perform or disappointment. In addition, a brief explanation of the concept of pacing as one possible intervention for Long COVID was added to the introduction of the manual, along with a note that pacing will not be part of the following sessions. An overview of all changes made to the intervention manual as a result of the focus groups is presented in Table 3. A summary of the intervention manual and its detailed changes in relation to the individual sessions is displayed in the S1 File.

**Table 3:** Overview of changes to the manual of the expectation management intervention of the SOMA.COV trial in response to patients’ feedback during focus group interviews.

Before focus groups	After focus groups
**Study description**	**Study description**
Description and procedure of the research project SOMA.COV	Addition of a statement on pacing as one treatment approach
Information on the development of Long COVID, its course and treatment approaches	
**“Vicious circle of fear”**	**“Vicious circle of protective behavior”**
Avoidance behavior as a consequence of dysfunctional and anxiety-related cognitions	Renaming of the “vicious circle of fear” and removal of the term “avoidance behavior”
**Behavior change exercise**	**Behavior change exercise**
Focus on avoidance behavior	Focus on testing negative expectations
	Specification of conditions (place, time, alone or with other people)
	Emphasis on open outcome

## Discussion

This study actively engaged patients in the development of an expectation management intervention for Long COVID, highlighting the importance of patient involvement in the development of treatments for this highly debilitating condition. The aims of the study were to explore the lived experiences, needs, and perspectives of patients with Long COVID regarding the intervention and to adapt the treatment manual accordingly. In line with the structure of the manual, topics discussed in the focus groups included the biopsychosocial model [9–11], cognitive restructuring of dysfunctional symptom expectations, an imagination exercise, the vicious circle of fear (acc. to [24]), a behavior change exercise, as well as further feedback and needs. A total of 13 themes were developed on the six topics, reflecting key aspects of lived experiences and patient perspectives.

Large parts of the manual received positive feedback by patients with Long COVID, including the psychoeducation on the biopsychosocial model, elements of cognitive restructuring and the imagination exercise carried out in the focus group session. Concerning the biopsychosocial model, the findings support the feasibility and acceptance of psychoeducation on biopsychosocial interactions in Long COVID. In particular, feelings of disappointment, insecurity and depressed mood were mentioned as both consequences of somatic symptoms as well as influencing somatic symptom burden. The impact of social aspects related to Long COVID on physical and mental wellbeing, including difficulties with the health care system, insurance, and financial problems, were considered particularly important by patients. These experiences are consistent with those reported in other qualitative studies on Long COVID [39]. In a systematic review of qualitative findings on the lived experiences of people living with Long COVID, Hossain et al. [26] emphasize the critical impact of financial stress and challenges within the health care system on the wellbeing of affected patients. In our study, many participants additionally pointed to difficult family situations as another important social aspect contributing to the biopsychosocial experience of their disease. Wishes to address challenges posed by changes in social roles as part of the intervention were also expressed. While providing patients with strategies to cope with their changed social role and to communicate their condition to friends and family seems to be important, the inclusion of such strategies was beyond the scope of an expectation management intervention focused on the modification of illness-related anxiety and dysfunctional symptom expectations. The same applies for support in dealing with financial hardship or conflicts with insurances, pointing to the need for appropriate counseling and support services. Regarding cognitive restructuring, many participants reported to benefit from developing functional thoughts and considered it a helpful tool for coping with symptoms. Accepting both the symptoms and having to adapt to a lower activity level were also perceived as helpful in reducing mental and physical stress. This is in line with reports of other qualitative studies on the needs of patients with Long COVID which identified “mindset changes” through acceptance and optimism as important coping mechanisms [39]. Promoting acceptance in dealing with symptoms was not the focus of the present mechanism-based intervention. However, given the coping experiences described above and the growing evidence for acceptance-based interventions (e.g., acceptance and commitment therapy) for reducing distress and improving quality of life as well as psychological flexibility in chronic health conditions [40,41], this appears to be a possible direction for further interventions [42].

Feedback on the vicious circle of fear was divided. On the one hand, several patients could relate their own experiences of fear-avoidance behavior to the model, while others considered the avoidance of activities a result of a “rational assessment”, denying emotional involvement. These patients perceived avoidance and safety behavior not as a dysfunctional process, but as necessary in order to adapt to the recurrent worsening of symptoms after exertion. This position may be partially explained by patients’ frequently reported experience that Long COVID symptoms are trivialized, not least by health care providers [39,43], which may result in reluctance to engage with the idea of anxiety being a maintaining factor of somatic symptoms. Several studies report patients’ experience of somatic symptoms being dismissed or considered purely as a mental health issue [43–45], especially patients with previous mental health illnesses [39]. At the same time, not experiencing accompanying emotions of avoidance or safety behavior could also hint towards reduced emotional perception in a subgroup of Long COVID patients. Since prior studies repeatedly confirmed the association between emotion regulation deficits and persistent somatic symptoms [11,46], emotion recognition and regulation is also worth investigating in patients with Long COVID.

When revising the intervention manual, we still included anxiety-related behavior as a maintaining factor of somatic symptoms, as anxiety is an important psychological risk factor of [12–14] and therefore part of existing CBT approaches for Long COVID [47]. In order to enhance the acceptability of the intervention, the vicious circle of fear was replaced by a “vicious circle of protective behavior” (adapted from Rief, 1998 [48]) as a model for explaining reinforcement of somatic symptoms (see Table 3). This vicious circle describes how perceived physical changes are interpreted as threatening and maintained through protective behavior, thereby shifting the focus from anxiety to the association between personal (protective) behavior and symptom persistence.

Concerning the behavior change exercise, some of the participants welcomed the rationale and found it supported their own experiences of overcoming fear and regaining self-confidence and self-efficacy after trying out previously avoided activities. However, some patients reported the impression that their worsening of somatic symptoms was more associated with behavioral endurance patterns characterized by unsteady activity levels or physical overuse. These contrasting experiences can be seen as a reflection of the insecurity many participants experience while trying out new behavior [49].

Suggestions for changes to the design of the exercise concerned removing the terms avoidance and safety behaviors, which several patients described as inducing pressure to “perform”. In revising the exercise, emphasis was placed on describing and testing dysfunctional expectations, without explicitly promoting a reduction in safety behaviors (see Table 3 and S1 File). Frequent feedback by the participants of the focus groups concerned activity pacing as an element missing in the preliminary intervention manual. Because of its symptomatic overlap with ME/CFS [50], pacing has been discussed as a coping strategy for Long COVID. Currently, research into the effects of pacing for Long COVID is limited [51]. While recent studies favor cautious exercise in Long COVID [52], the definition and delivery of pacing approaches for ME/CFS varies widely, including the question whether an increase in activity should be part of it. Consequently, meaningful conclusions cannot yet be drawn regarding its effectiveness in reducing somatic symptom burden [53], which is why pacing was not included in the SOMA.COV intervention manual. At the same time, pacing approaches seem to go along with high acceptance among those affected by Long COVID. Future research should investigate clearly defined pacing approaches for Long COVID in order to inform further intervention development.

Our sample predominantly consisted of middle-aged women, which aligns with existing literature indicating that women are more frequently affected by Long COVID than men, particularly individuals in middle and older age groups [5]. Additionally, participants displayed a considerable impairment of their ability to work, prolonged illness duration, and a high overall symptom burden, also consistent with previous findings [2,54]. These aspects show commonalities with Long COVID patients in other studies and emphasize functional impairment of those affected.

## Strengths and limitations

This study actively involved patients in the development of an expectation management intervention for Long COVID. Instead of evaluating the treatment manual after its implementation, it was adapted based on patient’s feedback before being applied in the three-armed randomized controlled trial SOMA.COV [23]. The use of different modalities for the focus groups (online and in person) allowed patients with varying degrees of impairment and different residences to participate. Almost half of the sample had experienced Long COVID symptoms for more than 1.5 years. Due to their prolonged experience with the condition, patients shared multiple individual coping strategies, which enriched their feedback on the intervention components.

However, selection biases must be taken into account when interpreting the results. First, a significant proportion of the sample was recruited from the psychosomatic outpatient clinic at the UKE, i.e., patients who have already actively presented in a psychosomatic setting, meaning that the perspectives of individuals who are critical of biopsychosocial interactions or of those who are too impaired to participate in a three-hour focus group may be missing. Similarly, the delivery of the material in the focus groups required a high degree of cognitive effort and perspective-taking. Given that concentration difficulties are a common symptom of Long COVID [55], the voices of those less impaired may be overrepresented.. Furthermore, most participants had attended, and some continued to attend, a Long COVID group therapy including elements of CBT. We did not control for additional therapy experiences, which may have influenced the individual use and approval of specific CBT concepts as well as the perspectives on the intervention manual presented. Also, all participants had a high level of education and were fluent in German language, which may have contributed to a better understanding of the content provided. In light of less educated groups being disproportionately affected by Long COVID [56] as well by persistent somatic symptoms in general [11], this limitation should be considered. Finally, the results were not shared with the participants, which prevented further discussion and validation of the findings by those involved.

## Conclusion

In the present qualitative focus group study, patients with Long COVID provided positive feedback on an expectation management intervention while also highlighting important adaptations necessary for this patient group. Our results indicate that both patients and health care practitioners should be better educated about the importance of biopsychosocial interactions and the role of psychological factors in the persistence of somatic symptoms to reduce stigmatization. Given the novelty of the condition, patients’ experience of stigmatization, and insecurities regarding the application of treatment concepts for which evidence to date is insufficient, involving patients with Long COVID in qualitative evaluations of interventions is essential to gain a better understanding of their needs and to better tailor future interventions.

## Supporting information

S1 File Summary of the expectation management intervention manual of the SOMA. COV trial including incorporated changes in response to the focus groups. (DOCX)

S2 File Interview guide. (DOCX)

S3 File COREQ checklist. (PDF)
