# Supplementary material for: Development of an expectation management intervention for patients with Long COVID: A focus group study with affected patients
Source: PLoS One. 2025 Feb 3;20(2):e0317905. doi: 10.1371/journal.pone.0317905 (PMC11790141; doi:10.1371/journal.pone.0317905)
Supplement: S1 File — (DOCX) [file pone.0317905.s001.docx]

**S1 File. Summary of the expectation management intervention manual of the SOMA.COV trial including incorporated changes in response to the focus groups**

Development of an expectation management intervention for patients with Long COVID:

A focus group study with affected patients

| Intervention manual before conducting focus groups | Changes to the manual after conducting focus groups |
| --- | --- |
| **Introduction material**   1. Study information:  - Procedure of the SOMA.COV study with explanation of the online format (four individual online sessions)  1. Long COVID:  - Information on its definition, development, course, and available treatment approaches | Addition of the concept of pacing as one further treatment approach:   - “Other approaches relate to symptom management, e.g. “pacing”. […] Patients report that pacing helps them manage stress and use their energy better.” |
| **1^st^ session “Living with Long COVID”**   1. Biopsychosocial model (**Topic 1**):  - Explanation of biopsychosocial interactions in chronic health conditions in general and Long COVID in particular  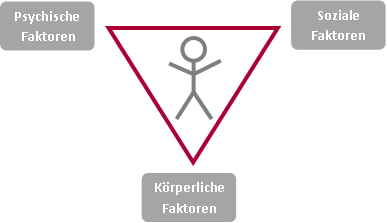 Psychological factors  Social  factors  Biological factors   - Illustration of the interaction between thoughts and physical reactions through the so-called “lemon exercise”:   “I would now like you to join me in imagining a plate with a beautiful, yellow, juicy lemon in front of you. […]”   1. Psychoeducation on expectations and illness-related anxiety 2. Structured recording of individual dysfunctional symptom expectations and illness-related anxieties |  |
| **2^nd^ session “Developing helpful thoughts”**   1. Cognitive restructuring (**Topic 2**):  - Psychoeducation on the interplay between thoughts, feelings and behavior, and their impact on bodily sensations - Development of an individual ABC model (**A**ctivating event, **B**eliefs, **C**onsequences) in order to restructure dysfunctional symptom expectations and illness-related anxieties  1. Imagination exercise (**Topic 3**), also available as an audio file for independent repetition: “Imagine how you feel when you feel really good, when you feel great. How would you notice this state most clearly? What would be different about your bodily sensations? […]” |  |
| **3^rd^ session “Tying out behavior (again)”**   1. Vicious circle of fear (**Topic 4**):  - Explanation of avoidance behavior in response to dysfunctional and anxiety-related cognitions   **External stimulus**  **Perception:**  Bodily sensations  **Emotion:** Anxiety, restlessness, feeling of loss of control  **Avoidance behavior:** e.g., cancel meetings and stay at home  **Stress response and increase in physical stress levels**  **Physical reactions** (e.g., breathlessness, pain)  **Focus of attention on somatic symptoms**  **Expectations/Thougths/Evaluations:** e.g., “If I overexert myself today, tomorrow will be ruined.”   1. Behavior change exercise (**Topic 5**):  - Planning of an activity (“behavior change exercise”) with focus on reducing avoidance/safety behavior:   “Avoidance behavior, that I no longer want to engage in” (Excerpt of the worksheet) | Vicious circle of protective behavior:   - Replacement of the vicious circle of fear and removal of the term “avoidance behavior”   **External stimulus**  **Perception:** Bodily sensations  **Protective behavior:** e.g., cancel meetings and stay at home  **Reduction in physical resilience**  Increase of **physical reactions** (e.g., breathlessness, pain) and of  **Focus of attention on somatic symptoms**  **Expectations/Thougths/Evaluations:** e.g., “If I overexert myself today, tomorrow will be ruined.”    Behavior change exercise:   - Focus on testing negative expectations:   “Which expectation/fear do I want to test?”   - Specification of conditions:   “What are the general conditions (when, where, with whom)?”   - Emphasis on an open outcome:   “What did I learn from this experiment?” |
| **Booster session**  Debriefing of the behavior change exercise  Repetition and deepening of sessions 1-3 |  |
