# Supplementary material for: Development of an expectation management intervention for patients with Long COVID: A focus group study with affected patients
Source: PLoS One. 2025 Feb 3;20(2):e0317905. doi: 10.1371/journal.pone.0317905 (PMC11790141; doi:10.1371/journal.pone.0317905)
Supplement: S2 File — (DOCX) [file pone.0317905.s002.docx]

**S2 File. Interview Guide**

Development of an expectation management intervention for patients with Long COVID:

A focus group study with affected patients

| Introduction  15min | - Welcome  - Introduction of the moderators and participants  - Presentation of the SOMA.COV research project  - Explanation of the role of participants: „Long COVID experts“, therefore co-development of an intervention in terms of participatory research  - Procedure: Discussion and exchange on planned sessions, execution of an exercise, breaks in between |
| --- | --- |
| **SECTION 1:**  Warm-up / Experiences and needs  10min | Which treatment helped/is currently helping you with your Long COVID symptoms? What are you missing? |
| **SECTION 2**:  Presentation and discussion of 1^st^ / 2^nd^ session of the SOMA.COV manual  25-30min | 1) **Biopsychosocial model**  - What do you already know about the connection between body and mind?  *Brief explanation with presentation of an illustration:*  *Body and mind interact closely, e.g., thoughts influence bodily sensations, and thoughts can be very "powerful".*  *Example:*  *Job interview coming up -> anxious thoughts -> stomach ache*  - What do you think about the model? What is your position on this?  2) **Cognitive restructuring**  *Education about dysfunctional symptom expectations and illness-related anxiety and their impact on medical treatments ( e.g., evidence for other medical conditions)*  *Examples:*  *“I expect that I won't be able to concentrate sufficiently to read a newspaper article.”*  *“I am afraid that the symptoms will never get better.”*  - How easy/difficult would it be for you to think of alternative thoughts?  - What alternative beliefs/thoughts come to your mind spontaneously? |
| **BREAK** 10min |  |
| **SECTION 3**:  Presentation and discussion of 2^nd^ session  25-30min | **Imagination exercise**  - How do you feel after the exercise?  - What was helpful for you? What changes do you propose? |
| **SECTION 4**:  Presentation and discussion of 3^rd^ session  20min | 1) **Vicious circle of fear**  *Brief explanation with presentation of an illustration:*  *"Vicious circle of fear" for illustrating avoidance behavior in response to anxiety and its maintenance*  - What do you think about the model? What is your position on this?  2) **Behavior change exercise**  *Education about avoidance behavior (withdrawal is understandable; however, it has been shown that it can be helpful to try out activities again)*  - In what way could you imagine conducting a behavioral change exercise?  - What would you see as possible pitfalls? What would be difficult for you? |
| **BREAK** 10min |  |
| **SECTION 5**:  Final feedback  25-30min | **Further feedback and needs**  What other changes to the intervention do you suggest? What are you missing in the manual? |
| **SECTION 6:**  Final round and acknowledgements  10min | - Thanks for participation  - Current emotional state of the participants |
